# Causative Pathogens in Endophthalmitis after Intravitreal Injection of Anti-vascular Endothelial Growth Factor Agents

**DOI:** 10.5041/RMMJ.10348

**Published:** 2018-10-04

**Authors:** Cecilia P. Labardini, Eytan Z. Blumenthal

**Affiliations:** 1 Department of Ophthalmology, Rambam Health Care Campus, Haifa, Israel; 2 The Ruth & Bruce Rappaport Faculty of Medicine, Technion–Israel Institute of Technology, Haifa, Israel

**Keywords:** Anti-VEGF, bacterial infection, endophthalmitis, intravitreal injection

## Abstract

Intravitreal injection of anti-vascular endothelial growth factor is currently the preferred treatment for several posterior segment diseases, including age-related macular degeneration and diabetic retinopathy, as well as macular edema and retinal vein occlusion. As an invasive procedure it involves risks. The most significant risk is infectious endophthalmitis, a sight-threatening and even a globe-threatening acute fulminant condition. Most common pathogens include *Streptococcus* and *Staphylococcus* species, surprisingly originating from the patient’s, surgeon’s, or nurse’s mouth. Infectious endophthalmitis may have devastating and irreversible effect, with *Streptococcus*-induced cases having the worst visual outcome. It is therefore crucial for clinicians to promptly recognize and treat such conditions, and, far more important, to put in place protective and preventive measures against this rare, but sight-threatening complication. To that end, this paper describes the most common pathogens causing endophthalmitis after IVI of anti-VEGF, and defines their source, to aid the physician in developing strategies to prevent this catastrophic infection.

## INTRODUCTION

Intravitreal injection (IVI) of anti-vascular endothelial growth factor (VEGF) has revolutionized in the past decade, and is currently the preferred treatment for several posterior segment diseases, among them age-related macular degeneration, diabetic retinopathy, as well as macular edema and retinal vein occlusion.^1^,^2^ Clinical trials have repeatedly and consistently demonstrated visual as well as anatomic improvement following therapy with anti-VEGF in the aforementioned diseases.^3^ Injections are usually administered at 1–2 monthly intervals for a period lasting between several months and up to many years.^4^ The use of these medications has increased considerably in the last 5 years,^1^ becoming the most commonly performed ophthalmic procedure in the United States.^5^,^6^

As in every invasive procedure, a risk of complications exists with IVI procedures, the most important one being endophthalmitis, which carries a devastating outcome.^1^,^7^,^8^ With the increasing number of IVI, the number of cases of endophthalmitis has also grown.^4^ Identifying the infectious agents involved in endophthalmitis after IVI is essential to provide the appropriate empiric antibiotic treatment.

Endophthalmitis is an intraocular inflammatory response to a bacterial or fungal infection inside the eye. Such infections start in the aqueous humor, the vitreous body, or both.^4^,^9^ Endophthalmitis is justifiably one of the most serious and urgent emergencies encountered in ophthalmic practice.^10^ Clinical signs and symptoms include decreased vision, mild to moderate pain, a red eye, anterior chamber inflammation, and vitritis.^11^

Even though the incidence of endophthalmitis after anti-VEGF injections is very low^4^,^11^ (rates range from 0.038% to 0.065%, which translates to 1 in 2,632 to 1 in 1,538),^8^ the visual prognosis is often poor, depending primarily on the time to treatment, the virulence of the infecting pathogen, and the treatment chosen. Prompt diagnosis and treatment can save eyes and achieve satisfactory visual results.^10^

This article describes the most common pathogens that cause endophthalmitis after IVI of anti-VEGF and defines their source, in order to provide strategies for preventing this catastrophic infection.

## PATHOGENIC AGENTS

Vitreous cultures are reported positive in 45%–60% of the cases of infectious endophthalmitis after anti-VEGF IVI,^1^,^4^,^11^ Gram-positive bacteria being responsible for over 95% of culture-positive cases.^4^

The most commonly isolated organisms in endophthalmitis following IVI are *Staphylococcus* (38%–60%) and *Streptococcus* species (25%–33%).^1^,^11^ Other less frequent causative organisms include *Bacillus* and *Haemophilus* species (Table 1).^1^

**Table 1:** Most Commonly Isolated Organisms in Endophthalmitis Following IVI.

Organism	Percent Found in Endophthalmitis
*Staphylococcus species*	38%–60%^1^,^11^
*Streptococcus species*	25%–33%^1^,^11^
*Bacillus species*	<10%^1^
*Haemophilus species*	<10%^1^

*Streptococcus* species are roughly 30% more frequent in endophthalmitis after anti-VEGF IVI than following incisional ocular surgery, compared to coagulase-negative *Staphylococcus* species, less frequently seen after IVI than after incisional surgery.^1^

There are no significant differences reported in endophthalmitis rates after the administration of either bevacizumab, ranibizumab, or aflibercept (anti-VEGF agents).^11^

An increased prevalence of oral flora-associated organisms, in particular *Streptococcus* species, in endophthalmitis following IVI compared to other penetrating intraocular procedures was found in multiple studies.^2^,^8^,^12^–^14^ These organisms are thought to originate from the physician, the assistant nurse, and/or the patient, secondary to speaking during the IVI procedure.^15^–^17^

Some studies found that wearing facemasks,^16^,^18^,^19^ and/or adopting a no-talking policy, reduced the speech-related bacterial contamination of the surgical field.^8^,^12^–^17^

One potential advantage of the use of facemasks is that it allows the patient, the physician, and the nurse assistant to speak during the procedure without the inherent risk involved. Of note, throughout the IVI procedure speaking is needed/encouraged to allow for the “pre-injection timeout,” and to verbally redirect the patient’s eye movements as needed, as well as to ease and comfort the patient during this potentially stressful procedure.^20^

Having said that, the use of facemasks during the IVI procedure is currently not considered to be part of standard of care, but, considering the increased risk of endophthalmitis resulting from oral species following IVI, its use, in addition to refraining from speaking and maintaining sterile conditions during the IVI procedure, was recommended.^21^

A study performed by Garg et al.^2^ demonstrated that a strict no-talking policy during IVI reduced the incidence of endophthalmitis post injection, presumably via a reduction in the oral cavity-associated spread of such pathogens. In this study two groups were compared. The first was a retrospectively examined group, in which a no-talking policy was not required: 0.057% of the cases (27/47,155 eyes) after IVI presented with endophthalmitis, of which 0.019% (9 eyes) had positive cultures. Of these positive culture endophthalmitis cases, 78% (0.015% of the total cases reviewed, 7 eyes) grew oral cavity flora including viridans streptococci, *Streptococcus mitis*, and *Lactobacillus*. Of the above, *Streptococcus* species represent more than 40% of oral cavity flora in adults,^22^ while *Lactobacillus* is often present in oral, vaginal, and gastrointestinal mucosa.^23^ The results were dramatically different when prospectively a no-talking policy was applied. In this arm of the study, containing 82,658 patients, only 0.024% of the cases (19 eyes) presented with endophthalmitis, 0.01% (8 eyes) had positive cultures, of which only 29% (2 eyes, 0.002% of the entire group) grew oral cavity bacteria, including a single case that cultured positive for *Streptococcus salivarius*/*vestibularis*, and one case in which *Streptococcus sanguinis* was isolated.^2^

The occurrence of culture-positive endophthalmitis cases while a strict no-talking policy was used illustrates that although a no-talking strategy is very effective, it will not eradicate all cases.^2^ Oral pathogens represent roughly 7% of isolates found when the normal human conjunctival flora is swabbed and cultured,^24^–^27^ hence this may reflect endophthalmitis independent of oral dispersion.^2^

Doshi et al.^16^ compared the no-talking policy to wearing a surgical mask via an interesting study design in which surgeons were asked to talk in front of blood agar plates. The surgeons were divided into four groups: the first did not wear a surgical mask; the second wore a surgical mask; the third did not wear a mask, and the culture plates were pre-exposed to 5% povidone-iodine; and the fourth one did not wear a mask, and a strict no-talking policy was implemented. The results showed bacterial growth as colony-forming units (CFU) per subject in each of the groups, respectively, as follows: the first group 8.8 CFU, the second group 1.1 CFU, the third group 0.1 CFU, and the fourth group 2.4 CFU. Statistically significant difference was found between all four groups, except for a non-significant difference (*P*= 0.115) between groups 2 and 4. This study suggests that both wearing a surgical mask and applying a no-talking policy significantly reduced the risk of endophthalmitis. These recommendations should be applied in addition to the already standard use of topical povidone-iodine prior to the injection.^16^

### Visual Prognosis

Endophthalmitis following IVI may have devastating long-term visual consequences.^1^

Endophthalmitis after intravitreal anti-VEGF injections, in which *Streptococcus* species are isolated, lead to poorer visual outcomes when compared to endophthalmitis caused by coagulase-negative *Staphylococcus* bacteria, as well as to culture-negative endophthalmitis. As many as 94% of endophthalmitis patients with *Streptococcus*-positive cultures had a final visual acuity (VA) of 20/400 (6/120) or worse. They were 125 times more likely to reach a low VA as compared to patients with culture-negative endophthalmitis, and 111 times more likely as compared to patients with coagulase-negative *Staphylococcus* endophthalmitis. There is no significant difference between culture-negative endophthalmitis and coagulase-negative *Staphylococcus* regarding visual outcomes,^1^ but the former tends to be associated with a less severe clinical course.^11^

### Culture-negative Endophthalmitis

Lack of growth of any pathogen may correspond to either a small bacterial load or suboptimal swabbing (both of which resulting in an inability to isolate and grow the pathogenic bacteria in culture), or to a truly non-infectious inflammatory process.^11^

While fibrin and the appearance of a hypopyon (the accumulation in the anterior chamber of white blood cells) are primarily associated with infectious cases, especially Gram-positive pathogens,^11^ non-infectious inflammation cases may also occasionally present with a hypopyon.^28^,^29^ Additionally, the onset of presentation post-IVI, the magnitude of vision decrease, and the amount of pain all tend to be milder in non-infectious cases, but are not diagnostic as they have been reported in both infectious^30^ as well as non-infectious cases.^28^,^31^ Hence, these findings are not reliable in differentiating true infections from sterile inflammation.^11^

### Aflibercept-related Inflammation

It is likely that at least some of the culture-negative endophthalmitis cases in fact represent sterile aflibercept-related inflammation.^28^,^29^ The American Society of Retina Specialists Therapeutic Surveillance Committee previously reported 15 cases of what appeared to be a sterile inflammation following intravitreal aflibercept (during the first 3 months after approval) originating from five separate drug lots. During this time 30,000 injections were applied, reflecting a sterile inflammation rate of 0.05% (15 eyes). From the cases reported, all but one case presented within a period of 3 days following injection. Pain was reported by 60%, while redness appeared in 40% of this group.^29^

Other studies reported cases compatible with non-infectious inflammation after aflibercept IVI, presenting symptoms within 1–3 days of the injection, with decreased or blurred vision and vitritis. Other symptoms such as pain and conjunctival injection were less frequent, and only few patients presented with a hypopyon.^11^,^28^ Those who present with what appears to be an aflibercept-related sterile inflammatory process have a good visual acuity prognosis following a time-limited course lasting 7–73 days.^11^

The differentiation between infectious and non-infectious endophthalmitis is extremely challenging; hence, injected individuals in whom a non-infectious endophthalmitis is suspected must be followed up very closely for the appearance of signs of improvement, or deterioration, when treated with topical steroids for the presumed diagnosis of a sterile inflammation.^11^

## PREVENTION

### Povidone-iodine

Evidence has shown that topical povidone-iodine is the most effective protective-prophylactic measure aimed at reducing the incidence of bacterial infection after IVI.^1^,^11^,^16^,^32^

A retrospective case-control series performed by Levinson et al. demonstrated that the application of povidone-iodine after placing the lid speculum decreases the incidence of endophthalmitis after IVI, due to the prevention of contact between the eyelid and the injection site.^33^

### Topical Antibiotics

Several studies consistently showed that the prophylactic application of topical antibiotics before as well as after the injection did not have any apparent beneficial effect on the rates of endophthalmitis post-IVI,^32^,^34^–^36^ and, surprising as it might seem, it may even increase the risk for endophthalmitis.^37^,^38^ This might be because multiple exposures to topical antibiotic drops alter the ocular flora and increase the presence of more virulent organisms (and perhaps assist in the appearance of resistant ones) on the ocular surface.^26^,^38^ These findings, in addition to the benefit of reduced cost, led the standard of care surrounding IVI to eliminate topical antibiotic prophylaxis.^37^

### Hand Antisepsis

Performing hand antisepsis at the beginning of all invasive procedures is important in order to reduce any bacterial load.^39^ When performing a session of injections, the hand antisepsis should initiate by washing them with soap or aqueous scrub to eliminate dirt and the usual bacterial load. Between each injection in the same session, alcohol-based rubs are ideal, due to their faster action and less skin irritation.^40^

### Gloves

Sterile gloves are required for performing aseptic procedures.^41^ Even though studies comparing the use of sterile and non-sterile gloves during IVI application have not yet being performed, this procedure should be considered an aseptic one, due to the fact that it involves penetration into an immune-privileged organ.^40^

## OTHER CONSIDERATIONS

The utilization of lidocaine gel (rather than in the form of drops) to anesthetize the surface of the eye, prior to the use of povidone-iodine antisepsis, was not shown to significantly alter post-IVI endophthalmitis rates.^42^ Similarly, the facility where the injection takes place (an operating room versus an outpatient clinic) was not shown to have any significant effect on the incidence of endophthalmitis.^37^

## CONCLUSIONS

Endophthalmitis after anti-VEGF IVI represents a grave visual outcome, so all efforts to reduce its incidence are justified. As IVI injections are carried out ever more frequently, it is imperative to understand the pathogenic process and identify underlying risk factors, and to study the etiological agents and factors involved, in order to properly prevent, or identify and treat, this vision-threatening condition. The most common causative agents are *Streptococcus* and *Staphylococcus* species due to an oral flora translocation from the patient, the surgeon, and/or the nurse assistant. In addition, one should always take into account the possibility of a sterile inflammation as well as other contamination pathways. Strict prevention, care, and follow-up after the procedure may help reduce post-IVI endophthalmitis rates.

## Abbreviations

CFU: colony-forming units
IVI: intravitreal injection
VA: visual acuity
VEGF: vascular endothelial growth factor
